# DNA-Directed Protein Anchoring on Oligo/Alkanethiol-Coated Gold Nanoparticles: A Versatile Platform for Biosensing Applications

**DOI:** 10.3390/nano13010078

**Published:** 2022-12-23

**Authors:** Ahmed Alsadig, Behnaz Abbasgholi-NA, Hendrik Vondracek, Barbara Medagli, Sara Fortuna, Paola Posocco, Pietro Parisse, Humberto Cabrera, Loredana Casalis

**Affiliations:** 1 Department of Physics, University of Trieste, 34127 Trieste, Italy; 2 NanoInnovation Lab, Elettra-Sincrotrone Trieste S.C.p.A., 34149 Trieste, Italy; 3 Optics Lab, STI Unit, The Abdus Salam International Centre for Theoretical Physics, 34151 Trieste, Italy; 4 Department of Medicine, Surgery and Health Sciences at the University of Trieste, 34149 Trieste, Italy; 5 Italian Institute of Technology (IIT), Via Melen–83, B Block, 16152 Genova, Italy; 6 Department of Engineering and Architecture, University of Trieste, 34127 Trieste, Italy; 7 Institute of Materials (IOM-CNR), Area Science Park, 34149 Trieste, Italy

**Keywords:** DNA-directed immobilization (DDI), gold nanoparticles (AuNPs), plasmonic immunoassays, thermal lens spectroscopy (TLS), biosensing, circulating disease biomarkers

## Abstract

Herein, we report on a smart biosensing platform that exploits gold nanoparticles (AuNPs) functionalized through ssDNA self-assembled monolayers (SAM) and the DNA-directed immobilization (DDI) of DNA-protein conjugates; a novel, high-sensitivity optical characterization technique based on a miniaturized gel electrophoresis chip integrated with online thermal lens spectrometry (MGEC-TLS), for the high-sensitivity detection of antigen binding events. Specifically, we characterized the physicochemical properties of 20 nm AuNPs covered with mixed SAMs of thiolated single-stranded DNA and bio-repellent molecules, referred to as top-terminated oligo-ethylene glycol (TOEG6), demonstrating high colloidal stability, optimal binder surface density, and proper hybridization capacity. Further, to explore the design in the frame of cancer-associated antigen detection, complementary ssDNA fragments conjugated with a nanobody, called C8, were loaded on the particles and employed to detect the presence of the HER2-ECD antigen in liquid. At variance with conventional surface plasmon resonance detection, MGEC-TLS characterization confirmed the capability of the assay to titrate the HER2-ECD antigen down to concentrations of 440 ng/mL. The high versatility of the directed protein-DNA conjugates immobilization through DNA hybridization on plasmonic scaffolds and coupled with the high sensitivity of the MGEC-TLS detection qualifies the proposed assay as a potential, easily operated biosensing strategy for the fast and label-free detection of disease-relevant antigens.

## 1. Introduction

The controlled immobilization of proteins on 2D or 3D substrates has shown great potential to revolutionize protein diagnostics and biomolecular recognition. Recent advancements in nanobiotechnology have enabled the development of strategies for the immobilization to 2D and 3D surfaces of different biological molecules such as peptides, proteins, aptamers, and nucleic acids, allowing for the fabrication of multi-functional platforms for therapeutic and diagnostic purposes, including protein microarrays [1,2,3]. Yet, the major challenges associated with protein immobilization to a solid substrate concern the preservation of protein structure-function by avoiding structural alterations of the proteins during the immobilization process as well as the steric hindrance of the recognition sites [4]. Towards this end, alternative strategies to the direct, covalent linking of proteins are desirable. Specifically, the DNA-directed immobilization (DDI) of DNA-conjugated proteins provides a promising versatile approach: it combines the advantages of the self-assembling of short single-stranded DNA oligos on surfaces with the facile sequence-specific hybridization with complementary DNA strands conjugated with specific proteins and/or protein binders: by conjugating different proteins with different oligo sequences, their respective immobilization on a DNA microarray of complementary strands is straightforward, allowing the easy transformation of a DNA microarray into a protein microarray by Watson–Crick hybridization [5,6,7]. In contrast to the covalent attachment, biomolecules tethered through DDI have shown full retention of the functionality as they are anchored onto the substrate via DNA spacers. Moreover, utilizing DNA strands as spacers between the substrate and the biomolecule of interest reduces the steric hindrance and enhances the chances for biomolecular recognition. However, while DNA grafting and DDI are well-known techniques for the immobilization of proteins on 2D substrates, they have not been exploited in association with 3D gold nanostructures, essentially due to the low sensitivity of plasmonic detection when the AuNPs are decorated with nm-size molecules. In particular, the reproducible, density-controlled functionalization of complex surfaces with DNA linker strands constitutes one of the major issues for DDI applications.

In the framework of biosensing, several groups have ingeniously demonstrated the utility of the DDI approach. For example, N. Tort et al. reported a strategy employing bio-functional plasmonic nanostructured chips manufactured on a DNA microarray to detect anabolic-androgenic steroids (AAS) through the binding with hapten-oligonucleotides, i.e., small molecule-oligonucleotide conjugates, detected by localized surface plasmon resonance (LSPR) [8]. In our group, Deka et al. developed a colorimetric assay for monitoring helicase activity using DNA conjugated to AuNPs and promoting the formation of DNA-AuNP aggregates through the addition of DNA linkers [9]. The desegregation of the particle assemblies promoted by the helicase activity caused a change of their optical properties, as monitored through UV/Vis spectroscopic absorbance measurements. Changes in the disassembling kinetics are related to the enzymatic activity and can be used to screen specific inhibitors of the enzyme. 

**Figure 1:**
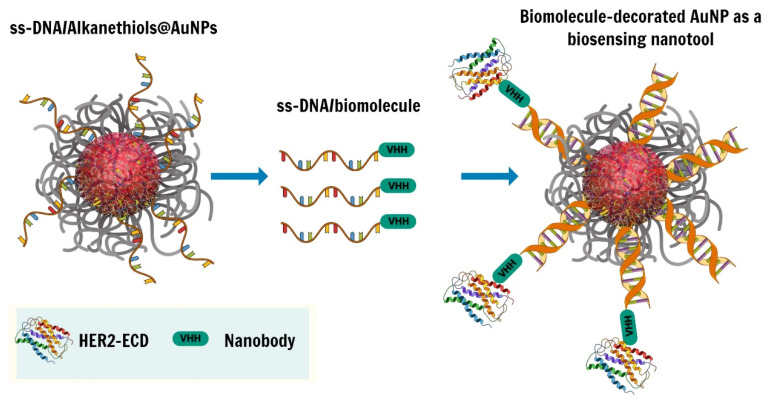
Schematic representation of the DNA-directed immobilization (DDI) strategy used in this work.

Herein, we propose for the first time to load protein-DNA conjugates through a DDI approach on ssDNA-AuNPs. Clearly, when coupled to a sensitive detection technique, this offers a great opportunity to construct a multifunctional platform that can be geared to a wide range of applications ranging from bioimaging to biosensing, gene regulation, and drug delivery. As a first step, we set out to implement the DDI approach utilizing and optimizing the parameters of a mixed self-assembled monolayer of: thiolated ssDNA moieties; bio-repellent thioalkylated oligo-ethylene glycol (OEG) molecules (specifically, a top-terminated (OEG)_6_ undecanethiol, named TOEG6) following the protocol developed by Deka et al. An optimized amount of ssDNA molecules were in fact needed to guarantee the active loading of the DNA-protein conjugates and to avoid steric hindrance effects, while the OEG-terminated molecules prevent the aspecific binding of proteins/small molecules on the particle surface. Specifically, in the framework of cancer-associated antigen detection, we chose to work with ssDNA-conjugated nanobody fragments, referred to as C8, optimized for the quantitative detection of the extracellular domain of the HER2 antigen, highly expressed in many types of cancer, including HER2 positive breast cancer (Figure 1). To overcome the limitations of SPR detection and to guarantee an improved sensitivity of the assay, we then capitalized on a novel approach based on a miniaturized gel electrophoresis chip (MGEC) integrated with online thermal lens (TL) detection, detailed in [10,11]. With this technique, 100-fold more sensitive than standard SPR, the measured variable is the change of the refractive index (enhanced by the presence of the gel matrix) associated with the system temperature change induced by the energy-to-heat conversion of light adsorption at the plasmonic resonance of the AuNPs. We already demonstrated the possibility of quantifying the number of thiolated DNA strands loaded on the AuNP surface in a mixed monolayer of different relative concentrations of ssDNA and TOEG molecules [11]. In the following, we will show that this implementation also allows for the rapid, sensitive, and straightforward detection of DNA-conjugated protein binders loaded onto the AuNPs through DDI and subsequently for the biorecognition of Her2-ECD. 

## 2. Materials and Methods 

### 2.1. Materials

Gold (III) chloride solution, Trisodium citrate dihydrate, monoclonal anti-polyhistidine−peroxidase antibody anti-His (HRP Sigma #A7058), and (Ethylenedinitrilo)tetraacetic acid (EDTA) were purchased from Sigma-Aldrich Chemical Co. (St. Louis, Missouri, US). The oligonucleotides used in this study were purchased from Biomers GmbH (Germany) referred to as, cF9-SH: 5′-CTTCACGATTGCCACTTTCCAC-3′ with 5′-thiol C6 modification (22 bases) and F9-maleimide: 5′-GTGGAAAGTGGCAATCGTGAAG-3′ with 5′-maleimide modification (22 bases). Phosphate-buffered saline (PBS) was purchased from “Thermo Fisher Scientific Inc., U.S.”, (Waltham, MA, USA). All the lyophilized DNA sequences were dissolved in Tris 10 mM, EDTA 1 mM, pH 8.0 (TE buffer) at a concentration of 100 µM for cF9 and 1 mM for F9. They were subsequently aliquoted, and then stored at −20 °C. Nanobodies (single domain nanobodies or VHHs) referred to as C8 [12,13] were produced with a terminal Cys-tag as described in [14]. The VHHs were aliquoted and stored at −20 °C. 1 mercaptoundec-11-yl)hexa(ethylene glycol) (SH-(CH_2_)_11_-EG_6_OH) (for brevity, TOEG6) was purchased from Prochimia Surfaces (Gdynia, Poland); Human HER2/ErbB2 protein, His-tagged was purchased from Acro Biosystems (Newark, Delaware, US). The buffer used in this study was made of 10 mM phosphate buffer (pH 7.0) prepared in Milli-Q water. Enhanced chemiluminescent substrate (ECL) reagents were purchased from Euroclone (Pero, Italy) and used for chemiluminescence.

### 2.2. Synthesis and Characterisation of AuNPs

Gold nanoparticles were synthesized by sodium citrate reduction of hydrogen tetrachloroaurate (HAuCl_4_) as described by Turkevich et al., with some modifications [15]. Prior to the synthesis, all glassware was thoroughly cleaned in aqua regia, rinsed several times with Milli-Q water, and left to dry overnight. In a 250 mL 3-neck round bottom flask (RBF), 1.095 mL of 17.3 mM HAuCl_4_ was added to 43.6 mL of Milli-Q water and brought to boil with vigorous stirring on a magnetic stirrer hotplate. Then, 300 μL of 245 mM of trisodium citrate dehydrate was added rapidly. The yellow solution turned to ruby red within a few minutes. To ensure the complete reduction of the gold salt, the stirring was continued for additional 20 min. The resulting gold colloids were protected from light and stored at 4 °C until used. The morphology and the average diameter of AuNPs were verified by transmission electron microscopy (TEM) and atomic force microscopy (AFM), while the optical properties of the AuNPs were characterized by UV-Vis spectroscopy.

### 2.3. ss-DNA/TOEG6@AuNPs Functionalization

A low pH assisted method was used to modify citrate-capped AuNPs according to a published protocol with slight adjustments. Briefly, cF9- SH (5.9 μL of 100 μM stock) was added to the AuNPs (750 μL of 2.6 nM stock) then mixed by vertexing and left to react at room temperature for 10 min. Next, HCl-citrate buffer (1.5 mL of 10 mM stock, pH 4.3) was added to neutralize the negative charges of the DNA backbone. The solution was then incubated for 30 min, allowing the thiols at the termina of the DNA strands to form bonds with the AuNP surface. Next, TOEG6 (17 μL of 300 μM stock) was added to the mixture, vortexed and left to react for 10 min to passivate the remaining surface. Nanoparticles were then purified by centrifugation (2X, 14,462× *g*, 15 min each in Milli-Q water). The final pellet was redispersed in either 1.0 mL of 10 mM phosphate buffer (pH 7.0) or 500 μL of 10 mM Saline phosphate buffer (100 mM NaCl, pH 7.0) as a hybridization buffer.

### 2.4. Assessing the Conformation of DNA Strands on AuNP Surface 

The possible configurations of DNA on the AuNP surface were assessed using a Zetasizer Nano ZS dynamic light scattering system (DLS) (Malvern Panalytical, Cambridge, UK). Particles were prepared as described previously and dispersed in 10 mM phosphate buffer (pH 7.0). For preparing ds-DNA/TOEG6@AuNPs, ss-DNA/TOEG6@AuNPs were dispersed in the hybridization buffer. Then, the complementary sequence was added to the particles in 10x excess, gently mixed, and kept at room temperature for an hour. Unbound DNA strands were then removed by centrifugation (2X, 14,462× *g*, 15 min) and the resulting samples were finally dispersed in the same buffer; 500 μL of each of the samples was used for DLS analysis.

### 2.5. Stability of ss-DNA/TOEG6@AuNPs in a Biological Environment

AuNPs and ss-DNA/TOEG6@AuNPs were prepared and redispersed in a final volume of 500 μL of diluted serum (1:1) in Milli-Q water. Samples were incubated for 0.5, 4, and 24 h at room temperature and then analyzed spectrophotometrically in a range from 400 to 800 nm. A baseline correction using diluted serum with identical concentration was used.

### 2.6. Biofunctionalization of AuNPs with Anti-HER2 Nanobodies

The conjugation of C8 biomolecule to a DNA strand (F9-maleimide) was conducted through a maleimide reaction as presented in Figure S1 (Supplementary Materials). Nanobodies contain cysteine in C-terminal with a -SH group (thiol group) that permits the reaction with the maleimide group present in the single strands (F9-maleimide). For the conjugation protocol, the nanobodies were first diluted in HEPES buffer 10 mM pH 7.4 at a concentration of 100 µM and the reducing agent TCEP was added in 10-fold molar excess. This solution was left for 20 min at room temperature. The F9-maleimide (concentration of 1 mM) was added to the solution of the nanobodies with a molar ratio ssDNA: protein of 10:1. The reaction was conducted in HEPES buffer 10 mM pH 7.0. Controlling the pH is a crucial step to avoid cross-reaction between amines and the free thiol group of cysteine. This solution was then incubated for two hours at room temperature. The conjugates were purified using G-25 Illustra Microspin Columns (GE Healthcare Life Science, Italy). Finally, the conjugates were aliquoted to avoid detrimental freezing/thawing cycles and stored at −20°C. Target DNA strands conjugated with C8 nanobody referred to as C8-F9 were introduced to the ss-DNA/TOEG6@AuNPs nanosystem with a concentration of 100 nM. After an hour of incubation at room temperature with moderate shaking, the resulting NPs were characterized with UV-Vis spectroscopy as well as gel electrophoresis. 

### 2.7. Dot Blot Immunological Assay for Detection of HER2 Antigen

Dot blot was employed to test the binding between the anti-HER2 nanobody and the HER2-ECD antigen. To accomplish this, 3 μL of C8-labelled AuNPs was slowly spotted on the center of a grid drawn on nitrocellulose membranes (3 × 3 cm^2^), stored for drying, and blocked with 5% bovine serum albumin (BSA) in PBST (1X PBS buffer + 0.01% tween) at room temperature for 60 min to saturate aspecific binding sites on the membranes. Next, the saturated membranes were incubated at room temperature for 60 min with 1:1000 diluted His-tagged HER2 (C_Initial_ = 0.44 mg/mL) in PBS. The membranes were then washed with PBST buffer (3 times, 5 min each) and incubated with 1:2500 diluted anti-His antibody conjugated with HRP (C_initial_ = 50 U/mL) for 60 min at room temperature, washed thrice with PBST buffer (once for 15 min, 2 times for 5 min each), followed by incubation with ECL western blotting reagent for 2 min. Finally, the membranes were developed with chemiluminescence and analyzed using an ImageQuant™ LAS 4000 digital imaging system (GE Helthcare, Chicago, IL, U.S.) and the corresponding software. Membranes spotted with control samples, and C8-AuNP conjugates were prepared following the same protocol.

### 2.8. Detection of HER2 Using Online TLS Coupled with MGEC

The fabrication of the MGEC, the TL setup, and the optimization of the system were reported previously [11]. For the quantitative detection of the different amounts of HER2-ECD in solution, the channel filled with agarose gel (100 μL of 0.5% agarose in 0.5 × TBE buffer (Tris-borate-EDTA)) was boiled in a microwave for 20 s. Then, 100 μL of gel was loaded into the channel. The polymerization process lasted 5 min. Next, HER2-ECD (from 5 nM to 50 nM) was incubated with C8-decorated AuNPs for 60 min at room temperature before being subjected to analysis. Afterwards, the buffer (40 μL) and the sample (dispersed in the buffer, 40 μL) were carefully loaded into the waste reservoir (WR) and sample reservoir (SR), respectively, for 5 min; 150 V was applied to the MGEC through platinum electrodes. After each run, the gel was removed from the channel using a hot water bath.

## 3. Results and Discussion

### 3.1. Characterization of Synthesized AuNPs

**Figure 2:**
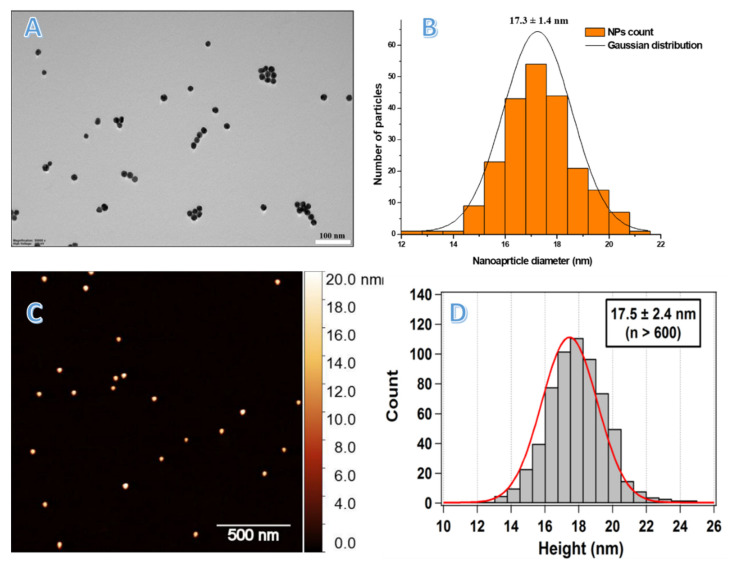
(**A**,**B**) A TEM micrograph of synthesized AuNPs with a scale bar of 100 nm, and the statistical analysis indicating nanoparticle size distribution evaluated for over 250 nanoparticles. (**C**,**D**) A 2 × 2 μm^2^ AFM non-contact mode image of AuNPs deposited on poly-L-ornithine-coated mica, and its corresponding statistical analysis.

Citrate-capped AuNPs were synthesized based on a standard wet chemistry approach in which sodium citrate acts as a reducing and protective agent as reported elsewhere [16]. A characteristic surface plasmon resonance (SPR) band of prepared AuNPs exhibited a maximum absorption at 520 nm as shown in Figure S2, consistent with the typical SPR band of AuNPs, confirming the formation of particles. This was further confirmed by TEM micrographs, which revealed an average particle diameter of 17.3 ± 1.4 nm (Figure 2A,B). Additionally, AFM was used for characterizing the particles: although not commonly used in the NPs field, sample preparation for AFM is faster than for TEM and the analysis can be performed in air as well as in a liquid, physiological environment. We established a procedure to immobilize AuNPs on mica, a natural material that due to its very flat surface (roughness ≤ 0.3 nm) is particularly suitable for AFM imaging. Specifically, we used mica coated with poly-L-ornithine (PLO), a polymer containing amine groups, which enables the electrostatic binding of the negatively charged, naked AuNPs. In this way, the displacement of the particles during AFM scanning is avoided (see details in Figure S3). The same polymer is used to bind DNA on surfaces through the phosphate backbone and is used in the literature for the AFM imaging of DNA both in dried and in liquid conditions [17]. By optimizing the relative concentrations of the PLO and AuNPs, a simple, fast, and reliable method based on a firm attachment of AuNPs for AFM imaging could be established. The AFM measurements yielded a value of 17.5 ± 2.4 nm, which is in agreement with the TEM analysis reported above (Figure 2C,D). 

### 3.2. Mixed-SAM Formation on the AuNP Surface 

**Figure 3:**
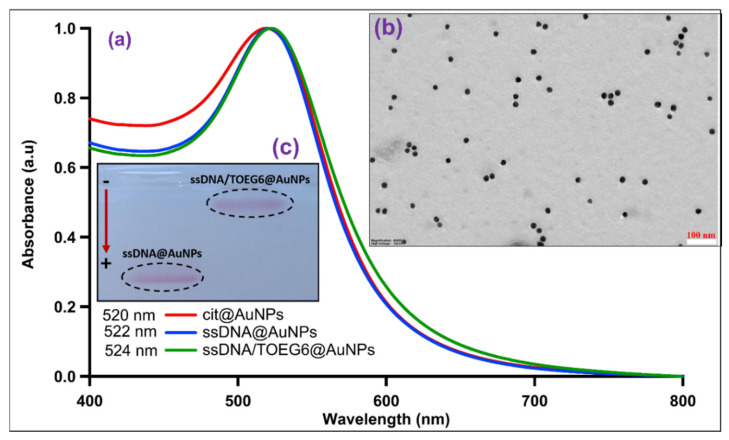
(**a**) The UV/Vis absorption spectra of functionalized AuNPs at each step of mixed SAM formation; (**b**) TEM image of ss-DNA/TOEG6@AuNPs; (**c**) Gel electrophoresis digital photograph of AuNPs passivated with ssDNA and the mixed SAM.

**Figure 4:**
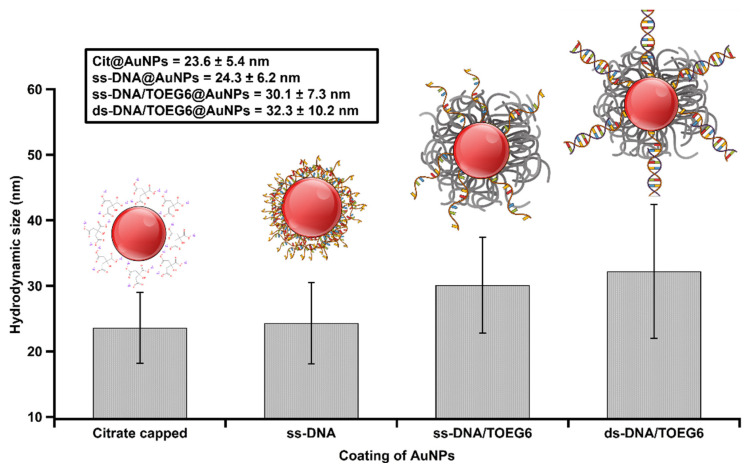
Summary of volume-weighted DLS measurements after each step of functionalization. The corresponding cartoons illustrate possible configurations of oligos adsorbed on the AuNP surface.

Citrate-stabilized AuNPs were functionalized with thiolated single-stranded (ss) DNA (referred to as cF9-SH) using the pH-assisted method [18]. This approach offers higher loading efficiency, and a single-step process that takes less than an hour compared to the 4–24 h and multiple steps required for salt aging. Then, we modified the DNA-AuNPs nanosystem by a post-treatment with a top-terminated oligo ethylene glycol alkanethiol, referred to as TOEG6, which acts as a spacing ligand. This molecule was selected because of its self-assembling properties: due to the van der Waals forces between the 11 CH_2_ groups along the alkyl chain, its assembly is far more efficient than the assembly of the DNA single strands (featuring only 6 C in the thiol linker and more pronounced steric effects due to the lateral dimension of the DNA strand vs. the alkyl chain). Based on its properties, TOEG6 is able to replace the unspecifically bound, thiolated ssDNA on the Au surface and then, as a function of exposure time, also the chemisorbed ssDNA. Moreover, its oligo-ethylene oxide sequence confers colloidal stability in aqueous solutions, which includes physiological and biological media. The ss-DNA/TOEG6 mix-SAM also possesses bio-repellent properties and allows for rational control over the DNA probe surface density, paving the way to construct more sophisticated functional architectures for various applications in biosensing [19]. The conjugates were characterized by UV/Vis spectroscopy, TEM, and gel electrophoresis (Figure 3). Our measurements revealed the formation of highly stable functionalized particles. The modified AuNPs were shown to migrate in the gel, showing a retardation of the ss-DNA/TOEG6@AuNPs band compared to ss-DNA@AuNPs. The delay was attributed to a combination of the size increment and the reduced number of DNA strands replaced by the neutral TOEG6 molecules. Based on fluorescence measurements, we estimated a coverage of 42 strands per AuNP in the working conditions specified in the materials and methods section. The DLS measurements of the AuNP hydrodynamic diameter (D_H_) evolution were used to determine the possible configuration of the DNA strands adsorbed on the AuNPs, as shown in Figure 4. At each functionalization step, the D_H_ of the AuNPs increases progressively, starting from the average particle diameter of the naked particles of 23 ± 5 nm. It is crucial to highlight that the D_H_ obtained by DLS takes into account the hydration shell that has the same translational diffusion coefficient as the particle. Therefore, due to the inherent working principle of this characterization technique, DLS reports significantly larger particle sizes than TEM or AFM. Interestingly, while D_H_ does not noticeably change upon DNA exposure, probably due to the mushroom configuration of the DNA SAM, a sensitive increase in D_H_ (~ 6 nm) was observed upon the introduction of TOEG6. It is plausible that the TOEG6 SAM domains caused the ss-DNA to have more rod-like conformation instead of the compact structure of the DNA monolayer around the particle [20]. The upright conformation of the DNA strands anchored on AuNP also increases the efficiency of the hybridization process. To verify that, the capture strands on the AuNP surface were hybridized with complementary target DNA. The duplex formation was confirmed by the 2 nm increase in the particle size attributed to the rigidity of ds-DNA with respect to ssDNA.

### 3.3. Assessing the Stability of ssDNA/TOEG6@AuNPs in a Physiological Environment

Next, we aimed to look at the stability of the ss-DNA/TOEG6@AuNPs and possible alterations in their optical properties. Several in vitro studies demonstrated that DNA-coated nanomaterials are prone to degradation in biological and physiological media [21,22,23]. Therefore, we performed our stability test by incubating AuNPs and AuNPs grafted with ss-DNA/TOEG6 with 50% diluted human serum for 0.5, 4 and 24 h. As expected, the incubation of the citrate-capped AuNPs with the serum resulted in a dramatic LSPR shift accompanied by a marked broadening of the spectrum due to nonspecific protein adsorption on the surface of the AuNPs (Figure S4). In contrast to this, in the case of ss-DNA/TOEG6@AuNPs, no broadening in the spectrum was observed, indicating that AuNPs remained highly monodispersed in the serum even after 24 h of incubation. It turns out that the presence of TOEG6 was able to effectively hinder the interaction between the AuNP surface and proteins in the serum, preventing aggregation in agreement with the previously reported findings of the excellent stability of such nanosystems in a high salt concentration environment [18]. 

### 3.4. AuNP Functionalization and ECD-HER2 Detection

Having demonstrated the stability of the system, we sought to explore its capabilities in the frame of cancer-associated antigen detection. We focused on a specific circulating biomarker known as the extracellular domain of the human epidermal growth factor receptor 2 (HER2-ECD). HER2 is a transmembrane protein that belongs to the epidermal growth factor receptors (EGFRs) family and is overexpressed in 15–30% of breast cancer patients [24,25,26]. HER2 overexpression (up to a 100-fold increase in HER2 receptors at the cell surface with respect to healthy tissue) is associated with accelerated growth, progressive metastatic disease, and a low disease-free survival rate [27]. This protein is composed of an extracellular domain (ECD), a transmembrane region, and an intracellular tyrosine kinase region [28]. The extracellular domain fragment of HER2 (HER2-ECD) is released into the bloodstream from the surface of tumor cells. As a result, HER2-ECD levels can be detected in the serum portion of blood, making it a minimally invasive and clinically useful biomarker for breast cancer [26,27].

**Figure 5:**
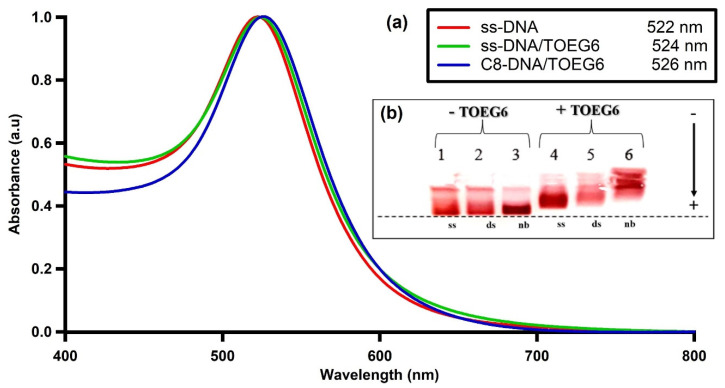
(**a**) The UV-Vis absorption spectra of VHH-terminated DNA/TOEG6@AuNPs at each step of the NP functionalization. (**b**) Gel electrophoresis of AuNPs functionalized with: (1) ss-DNA; (2) ds-DNA; (3) ss-DNA + cDNA-C8 conjugates; (4) ss-DNA/TOEG6; (5) ds-DNA/TOEG6; (6) ds-DNA/TOEG6 cDNA-C8 conjugates. The arrow indicates the migration direction.

**Figure 6:**
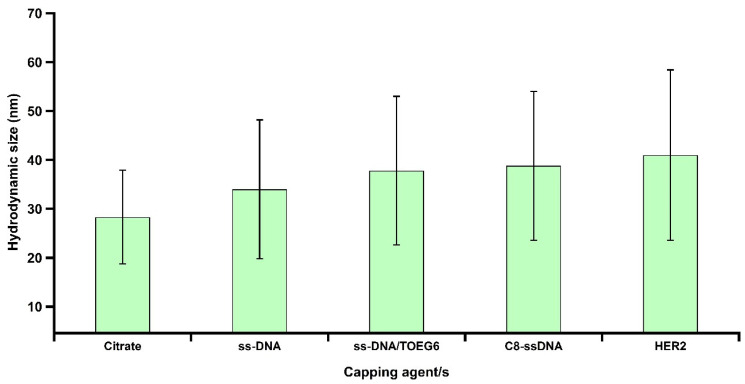
Volume-weighted DLS readouts showed that the resultant HER2 bioconjugation was achieved by the increment of D_H_ size from 38 to 41 nm for C8-terminated AuNPs and HER2@AuNPs, respectively, which can be attributed to the protein recognition.

In our previous work, we reported a strategy employing histidine-tagged HER2 (His-tagged HER2) immobilized on nitrilotriacetic acid (NTA) via cobalt (II) chelates, which can be used to optimize and personalize the treatment of HER2-positive cancer based on LSPR [16]. In this study, we concentrated on another important aspect of the assessment of the success of therapy: the quantification of an important biomarker associated with HER2 positive cancer. For doing so, we developed an assay that detects the HER2-ECD protein in solution by means of anti HER2-ECD nanobodies conjugated with DNA and linked via DDI to ssDNA/TOEG6 AuNPs (C8-terminated AuNPs). Nanobodies (Nbs) are the smallest antibody fragments (~15 kDa) that function as active antigen-binding units derived from the variable domains (VHH) of unique camelid heavy-chain antibodies (HC-Abs) devoid of light chains [29]. Nanobodies exhibit striking properties, including the small size, intrinsic stability, high affinity, and specificity that make them an important subject of study in a variety of research areas [30,31,32]. In this study, we used C8, a specific antibody fragment known to bind HER2-ECD to an epitope close to the one recognized by trastuzumab [12]. A concentration of 100 nM of C8-labelled DNA targets was selected to maximize the number of duplexes on the particle surface as well as to guarantee the interaction of the antigen with C8, which has shown a binding affinity of about 34 nM towards HER2-ECD [12,13]. As can be seen in Figure 5a, we observed a slight redshift in the SPR when the particles were hybridized with the C8-labelled DNA target, pointing to a successful conjugation of nanobodies to the particles. Figure 5b shows the photograph of a gel electrophoresis of particles at different steps of the surface modification. Clearly, the presence of TOEG6 (lane 4–6) allows for a better separation between the three conditions. Among all the bands, C8-terminated AuNP conjugates (lane 6) had the slowest migration, proving the presence of C8 nanobodies on the AuNPs. The DLS and dot blot immunoassay experiments were able to confirm this scenario. The DLS showed a sequential increase, even though small, of the average particle size (Figure 6). This increase extends towards the final step, the detection of HER2-ECD, indicating that the functionality of the C8 VHH is maintained when loaded onto the AuNPs.

**Figure 7:**
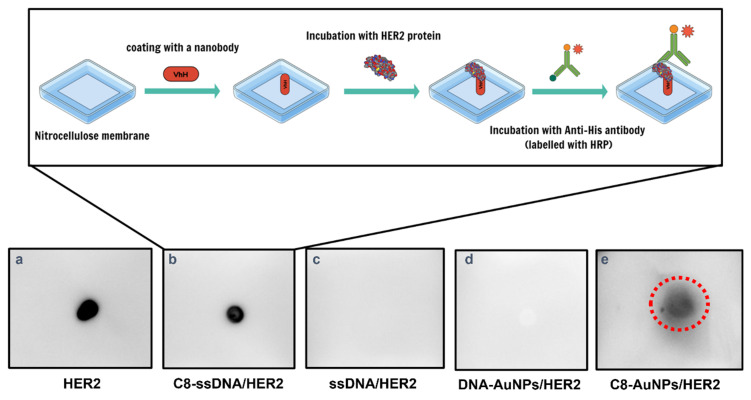
A scheme illustrating the preparation steps followed for dot blot immunological assay and the corresponding readouts on a nitrocellulose membrane in the presence of: (**a**) HER2; (**b**) C8-ssDNA/HER2; (**c**) ssDNA/HER2; (**d**) DNA-AuNPs/HER2; (**e**) C8-AuNPs/HER2. The red circle in panel e highlights the recognition ability of the immunocomplex to HER2-antigen.

This last observation was corroborated by immunological assays. The C8-conjugated ssDNA was immobilized on a nitrocellulose membrane, then soaked into His-tagged HER2-containing solution. After incubation, an anti-His antibody was employed to detect the formation of the immunocomplex. As shown in Figure 7, C8-conjugated ssDNA was detected upon the interaction with the antigen, indicating successful binding (C8-ssDNA/HER2). In contrast, no signal was observed for the ssDNA without labelling with the C8 nanobody (ssDNA/HER2). Next, the capability of forming a complex between gold nanoprobes and the target epitope was also evaluated by exposing, in a similar manner, C8-labeled AuNPs to the HER2-ECD, which also showed a signal, albeit less intense, indicating that the nanobodies linked to the AuNPs were capable of reducing the HER2-ECD protein in the assay solution down to a concentration of 6.2 nM (440 ng/mL). 

### 3.5. Novel Detection: MGEC-TLS

**Figure 8:**
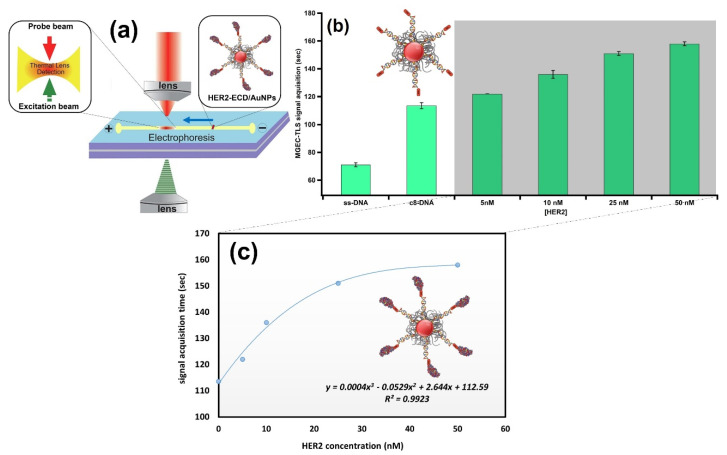
(**a**) A schematic illustration of the MGEC-TLS design employed for investigating HER2-ECD binding to C8-decorated AuNPs. The resulting immunoconjugates migrate toward the positive anode crossing the measurement point. (**b**) The MGEC-TLS signal acquisition time as a function of different steps of surface coating of AuNPs. The concentration of the antigen was varied from 5–50 nM. (**c**) Signal acquisition time curve of HER2-ECD measured with MGEC-TLS. The signal acquisition time is plotted versus HER2-ECD concentration; experimental data were fitted with a polynomial of third degree.

In order to overcome the detection limit of the assay, we used a miniaturized gel electrophoresis chip coupled with thermal lens spectroscopy (MGEC-TLS) to quantitatively relate the biorecognition of the immunocomplex to the mobility of the particles. Due to the high spatial resolution of the thermal lens detection system, along with the enhanced sensitivity [33,34] when coupled with gel electrophoresis, this combination has proven its robustness and potentiality as an advanced instrumental platform for designing biosensors in nanotechnology [10,11]. Herein, in order to obtain quantitative results, we measured the time required for the conjugates to reach the measurement point for the antigen concentrations ranging from 5 to 50 nM (350 to 3500 ng/mL), as depicted in Figure 8a. In line with our expectation, the time needed for the immunoconjugates to travel the required distance consistently increases with the quantity of HER2-ECD (Figure 8b). This resulted in a comprehensive trend that relates the concentration of loaded HER2-ECD to the MGEC-TLS signal acquisition time (the signal recorded when a sample passes the measurement point) (Figure 8c). The assay outperforms an assay previously presented in the literature, where Nbs are linked to a carbon-based screen-printed electrode (SPE) for the detection of HER2-ECD. The electrode assay was able to detect the antigen in a range between 1 and 200 µg/mL, yielding satisfactory results with a limit of detection (LOD) of 1 µg/mL [35]. Although electrochemical sensors were previously employed to detect HER2-ECD, in several cases reaching similar LOD as the MGEC-TLS (0.35 µg/mL)-based approach [36,37,38], the simplicity and the versatility of the proposed plasmonic immunosensor makes it very attractive. The immobilization of the target oligo-bound biomolecule obviates the need for using labels, reducing the operation complexity and assay costs. Furthermore, the incorporation of the newly developed system demonstrates online, real time signal response, and the capability of probing sub-μL volumes. Additional work will be required to improve the sensitivity of the nanosystem for buffer and serum sample applications at physiologically relevant concentrations.

## 4. Conclusions

Nanoparticles decorated with macromolecules are a promising platform for various potential applications in bionanotechnology and biosensing. In this study, we developed, characterized, and optimized an assay based on AuNPs functionalized with a mixed self-assembled monolayer of thiolated ssDNA and OEG-terminated alkanethiols for the controlled loading of protein-DNA conjugates. We then coupled the AuNP system with a novel optical detection technique, based on the monitoring of refractive index changes, which are induced in the proximity of the functionalized AuNPs upon biorecognition. The changes of the refractive index were amplified due to the higher enhancement factor of the gel in the MGEC. We demonstrated the validity of the approach in the frame of cancer-associated antigen detection. For this purpose, particles were labelled with a nanobody (C8), a specific antibody fragment to detect the presence of the HER2-ECD antigen in liquid. The C8-AuNP conjugates showed a noticeable change in LSPR. The DLS readouts revealed a noticeable change in the hydrodynamic size upon HER2 loading to Nb-terminated AuNPs, indicating a successful recognition. Our simple and rapid AuNP-based dot-blot immunoassay was used for quantifying the loading of HER2-ECD antigen down to a concentration of 440 ng/mL. We highlight here that with standard LSPR detection, the sensitivity was not enough to distinguish the binding of ECD-HER2 to the functional nanoparticles. Instead, the proposed MGEC-TLS approach coupled with DDI+DNA-AuNPs combines enhanced sensitivity and ease of preparation with low implementation costs, foreseeing a broad use of the assay for the detection of a wide range of analytes even in low or limited resource contexts. Though further verification is needed to ensure the biorecognition capability of the platform based on clinical samples, this study provides a methodology template that can contribute to the development of a multifunctional platform based on the DDI of proteins or protein fragments to AuNPs.

## Supplementary Materials

The following are available online at https://www.mdpi.com/article/10.3390/nano13010078/s1, Figure S1: Reaction of an SH-modified VHH with a maleimide-modified oligo leads to the formation of a stable nanobody-oligonucleotide conjugate, Figure S2.: UV/Vis spectrum and intensity-weighted size distribution (inset) of synthesized AuNPs, Figure S3.: Sample preparation used for Nanoparticle-AFM imaging, Figure S4.: Normalized optical spectra for AuNPs incubated with 1:1 diluted human serum at various incubation time. The aggregation seen in the optical profile (green) displays the aggregation behavior of the bare particles in the serum. Once coated with the mixed-SAM, a remarkable stability in the other optical spectra up to 24 h was observed.
