# Supplementary material for: DNA-Directed Protein Anchoring on Oligo/Alkanethiol-Coated Gold Nanoparticles: A Versatile Platform for Biosensing Applications
Source: Nanomaterials (Basel). 2022 Dec 23;13(1):78. doi: 10.3390/nano13010078 (PMC9823620; doi:10.3390/nano13010078)
Supplement: Supplementary file 1 [file nanomaterials-13-00078-s001.zip › nanomaterials-2106463-supplementary.pdf]

DNA-Directed Protein Anchoring on Oligo/Alkanethiol-Coated Gold Nanoparticles: A Versatile Platform for Biosensing Applications

Ahmed Alsadig [1,2,†], Behnaz Abbasgholi-NA [2,3,†], Hendrik Vondracek [2], Barbara Medagli [4], Sara Fortuna [5], Paola Posocco [6], Pietro Parisse [2,7], Humberto Cabrera [3,*] and Loredana Casalis [2,*]

1 Department of Physics, University of Trieste, 34127 Trieste, Italy
2 NanoInnovation Lab, Elettra-Sincrotrone Trieste S.C.p.A., 34149 Trieste, Italy
3 Optics Lab, STI Unit, The Abdus Salam International Centre for Theoretical Physics, 34151 Trieste, Italy
4 Department of Medicine, Surgery and Health Sciences at the University of Trieste, 34149 Trieste, Italy
5 Italian Institute of Technology (IIT), Via Melen–83, B Block, 16152 Genova, Italy
6 Department of Engineering and Architecture, University of Trieste, 34127 Trieste, Italy
7 Institute of Materials (IOM-CNR), Area Science Park, 34149 Trieste, Italy
* Correspondence: hcabrera@ictp.it (H.C.); loredana.casalis@elettra.eu (L.C.)
† These authors contributed equally to this work.

Supporting Information

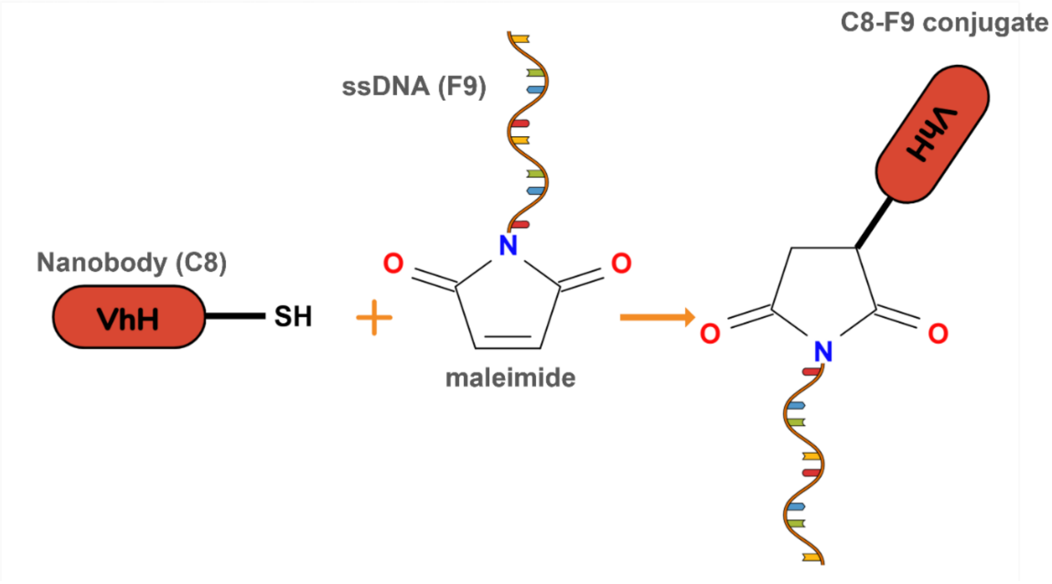

\

Figure S1: Reaction of an SH-modified VHH with a maleimide-modified oligo leads to the formation of a stable nanobody-oligonucleotide conjugate.

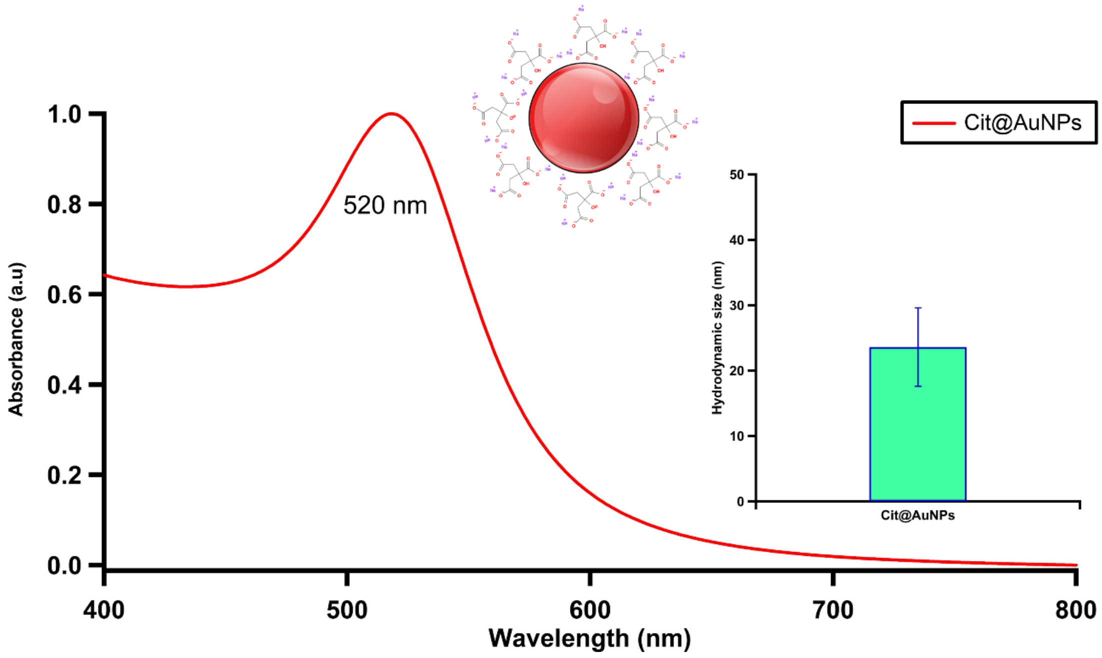

Figure S2: UV/Vis spectrum and intensity-weighted size distribution (inset) of synthesized AuNPs.

S3 Sample preparation for Atomic Force Microscope (AFM) imaging

Nanoparticle-AFM based image requires the particle to be rigidly anchored to AFM substrate which is virtually known to be mica. The deposition of AuNPs on AFM substrate was done as following; The mica substrate was freshly cleaved and directly coated with 10 µL of a 0.1 mg/µL of poly-L-ornithine solution (mol.wt 30000 – 70000, Sigma, St. Luis, MO, U.S.A). After 3 min of incubation, the surface of the mica was rinsed with 400 µL of Milli-Q and dried under the flow of nitrogen. 25 µL of sample suspension (diluted 1:50) was spotted on the mica modified surface, allowed to react for approximately 5 min, rinsed off with Milli-Q followed by nitrogen for completer dry. Imaging was recorded with Asylum research MFP-3D AFM operating in non-contact mode. The n-type silicon tip with 8 nm radius and cantilever with 110 ± 5 µm length, 32.5 ± 3 µm width, 1 ± 0.5 µm thickness, force constant of 1 N/m and resonance frequency of 90 kHz was used.

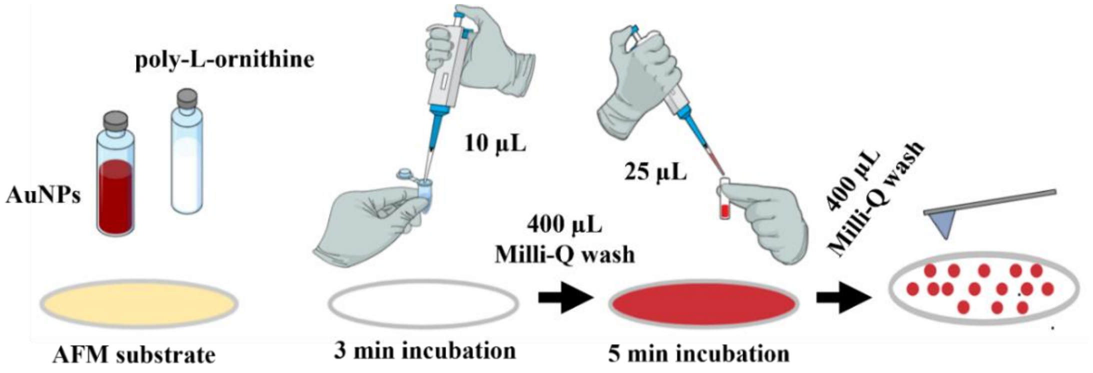

Figure S3: Sample preparation used for Nanoparticle-AFM imaging.

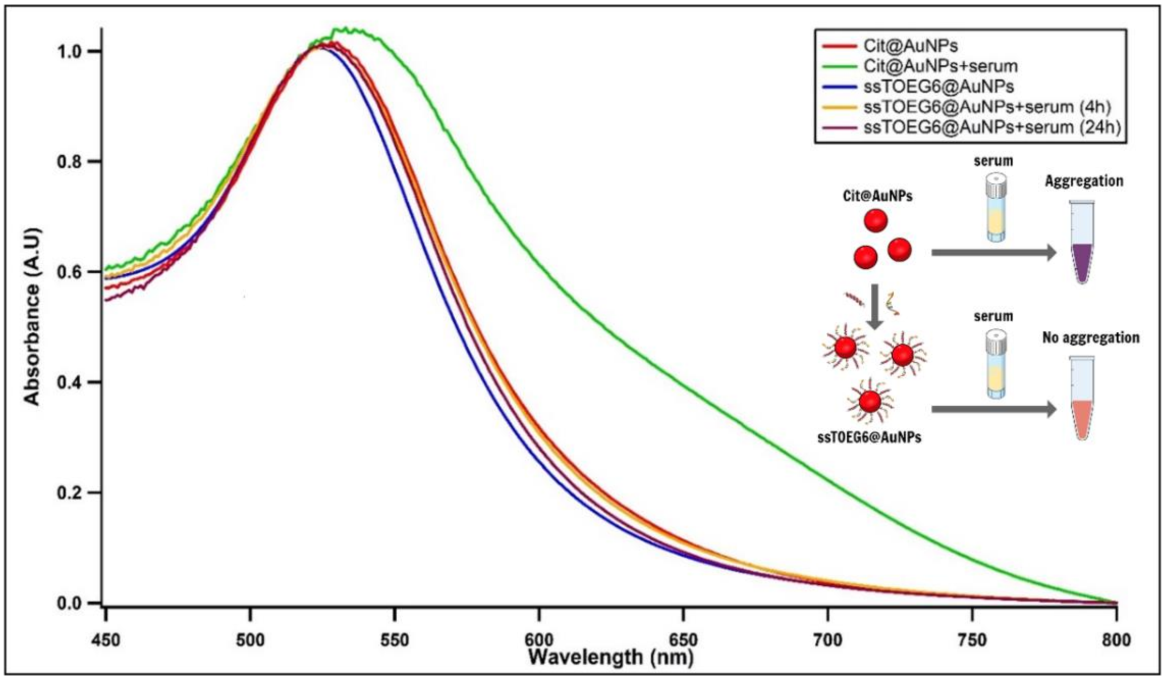

Figure S4: Normalized optical spectra for AuNPs incubated with 1:1 diluted human serum at various incubation time. The aggregation seen in the optical profile (green) displays the aggregation behavior of the bare particles in the serum. Once coated with the mixed-SAM, a remarkable stability in the other optical spectra up to 24 hours was observed.
